# Electrochemical H_2_O_2_ biosensor based on horseradish peroxidase encapsulated protein nanoparticles with reduced graphene oxide-modified gold electrode

**DOI:** 10.1186/s40580-020-00249-0

**Published:** 2020-12-16

**Authors:** Jeong-Hyeop Shin, Myeong-Jun Lee, Jin-Ha Choi, Ji-ae Song, Tae-Hwan Kim, Byung-Keun Oh

**Affiliations:** Department of Chemical & Biomolecular Engineering, Sogang University, Mapo-Gu, Seoul, 04107 South Korea

**Keywords:** Hydrogen peroxide, Horseradish peroxidase, Electrochemical, Reduced graphene oxide

## Abstract

In this study, an electrochemical biosensor composed of a horseradish peroxidase (HRP)-encapsulated protein nanoparticles (HEPNP) was fabricated for the sensitive and selective detection of H_2_O_2_. The HEPNP has a three-dimensional structure that can contain a large amount of HRP; therefore, HEPNP can amplify the electrochemical signals necessary for the detection of H_2_O_2_. Furthermore, reduced graphene oxide (rGO) was used to increase the efficiency of electron transfer from the HEPNP to an electrode, which could enhance the electrochemical signal. This biosensor showed a sensitive electrochemical performance for detection of H_2_O_2_ with signals in the range from 0.01–100 μM, and it could detect low concentrations up to 0.01 μM. Furthermore, this biosensor was operated against interferences from glucose, ascorbic acid, and uric acid. In addition, this fabricated H_2_O_2_ biosensor showed selective detection performance in human blood serum. Therefore, the proposed biosensor could promote the sensitive and selective detection of H_2_O_2_ in clinical applications.

## Introduction

Hydrogen peroxide (H_2_O_2_) is a very important in the fields of chemistry, biology, food studies, and in applications in clinical studies [1–3]. As a side product of some classic biochemical reactions catalyzed by enzymes, such as glucose oxidase, alcohol oxidase, lactate oxidase, and urate oxidase, H_2_O_2_ has been used to sense and track biochemical materials [4]. In the clinical and biological fields, H_2_O_2_ plays an important role in cellular mechanisms [5]. H_2_O_2_ is involved in reactive oxygen species (ROS) intracellular signaling as a mediator of several physiological processes, such as cell differentiation, proliferation, and cellular metabolism [6]. The high concentration of ROS, which is associated with oxidative stress, can directly damage mitochondrial DNA [7], cellular proteins [8], and lipids [9]. For these reasons, H_2_O_2_ has been an attractive target of research in the field of biosensors. However, its concentration is usually low in biofluids. For instance, the steady-state concentration of H_2_O_2_ in mitochondria was reported in the few-nano-molar (~ nM) scale [10]. Some approaches used to sense H_2_O_2_ have included fluorimetry [11], spectrometry [12], chemiluminescence [13], and electrochemistry [14]. Among these techniques, electrochemical sensing methods, which are based on the direct electron transfer between the enzyme and the electrode, are widely used because of their simple, rapid, and high specificity [15–17]. However, an electrochemical sensor using only enzymes, such as horseradish peroxidase (HRP), had low sensitivity and could only detect 0.3 mM of H_2_O_2_ [18].

To improve sensitivity, some researchers have used graphene oxide (GO) or reduced GO (rGO) because of its good properties, such as a large theoretical surface area, an extraordinary electrical character, good conductivity, durability, and many surface functional groups (hydroxyl, carboxyl, etc.) [19–21]. Because of these characteristics, the H_2_O_2_ electrochemical sensor could increase sensitivity; for example, HRP-palladium/GO could detect 25 μM [22], HRP/GO/silver could detect 25 μM [23], and HRP/GO/Chitosan/gold (Au) could detect 5 μM [24] in the linear range. However, its sensitivity was limited due to the low enzyme stability and two-dimensional enzyme immobilization, which could decrease the amount of enzymes fixed to the electrode. To overcome these disadvantages, recently, enzymatic nanoparticles have received attention because of their good properties in the field of enzymatic electrochemical detection. For instance, enzymatic nanoparticles can improve the stability of confined enzymes by protecting enzymes from denaturation, which can be carried out by external environmental conditions during analysis or storage [25]. Enzymatic nanoparticles can also increase catalytic activity by enlarging the surface reaction area and immobilizing a large amount of enzymes to the electrode with a three-dimensional structure [26, 27]. Due to these advantages, many research studies have used three-dimensional nanoparticles to improve sensitivity and increase the stability of enzyme-based electrochemical detections of H_2_O_2_. For example, the heme–iron(III) group-immobilized carbon sphere/SiO_2_/Au nanoparticle could detect 5 μM of H_2_O_2_ [28], the Chitosan/HRP/poly-L-DOPA nanoparticle could detect 1 μM of H_2_O_2_ [29], and the metal–organic framework/HRP cage and HRP onto mesoporous silica nanoparticle could detect 0.5 μM of H_2_O_2_ in a linear range [30, 31]. However, there were still limits in detecting a very low concentration of H_2_O_2_, so nanoparticles that cause a strong enzyme reaction were needed to increase sensitivity.

In this study, our group developed three-dimensional HRP-encapsulated protein nanoparticles (HEPNP), which could encapsulate very large quantities of enzymes. The nanoparticle, which was created by albumin protein and HRP, could hold a huge quantity of HRP in a three-dimensional structure, which can cause a powerful enzyme chemical reaction. By using these particles, a large amount of HRP could be attached to the Au electrode surface with a three-dimensional structure. In addition, the Au electrode was treated with rGO before fixing the particles to increase the surface area of the electrode for increasing the number of immobilized particles and improving electrode’s electrical property. In this paper, the synthesis of HEPNP was confirmed via transmission electron microscopy (TEM) and dynamic light scattering (DLS). Fabricated HEPNP/rGO/Au working electrodes were confirmed by field emission scanning electron microscope (FE-SEM) and cyclic voltammetry (CV). To investigate the electrochemical sensing performance of the fabricated biosensor used to detect H_2_O_2_, CV and amperometric i-t measurements were conducted.

## Methods/experimental

### Materials

Bovine serum albumin (BSA), glutaraldehyde, cysteamine, 0.01 M phosphate buffered saline (PBS, pH 7.4), 100 mM Tris buffer (pH7.4), 3,3′,5,5′-Tetramethylbenzidine (TMB), L-ascorbic acid (AA), uric acid (UA), human serum, N-methyl-2-pyrrolidone (NMP), and rGO were purchased from Sigma Aldrich (USA). A bare Au electrode composed of gold (50 nm)/Cr (2 nm)/SiO_2_ was prepared by the National Nanofab Center (Korea), and 30% H_2_O_2_, 95% sulfuric acid (H_2_SO_4_) and pure ethanol (EtOH) were purchased from Daejung Chemical (Korea). HRP was purchased from Thermo Fisher Scientific (USA), and D( +)-glucose (dextrose anhydrous) was purchased from JUNSEI (Japan). Distilled water (DW) was purified through a Milli-Q system (USA).

### Synthesis of HEPNP

HEPNP was synthesized via previous method by using the ethanol desolvation process. First, 100 μL of 50 mg/mL BSA solution in DW was mixed to 20 μL of 25 mg/mL HRP solution in DW. Added to the solution was 400 μL of pure EtOH with a flow rate of 1 mL/min while being stirred at 850 rpm at 25 °C. The mixture’s color then turned opaque. For the crosslinking between BSA and HRP, 10 μL of 4% glutaraldehyde was added, and the solution was incubated for 12 h. After the reaction finished, a washing step was done by centrifugation (9000 rpm, 15 min), and the supernatant was removed. The protein pellet was dispersed by 500 μL of the washing solution (0.01% Tween 20 in 0.01 M PBS solution) three times. After the final centrifugation, the protein pellet was dispersed by 100 μL of 1% BSA in Tris-buffer to deactivate glutaraldehyde. The HEPNP was stored at 4 ℃ in a dark room. Its characteristics were analyzed by using transmission electron microscopy (TEM) (JEOL1010, Japan) and dynamic light scattering (DLS) (Zeta Sizer, USA) analysis.

### Preparation of HEPNP/rGO/Au working electrode for sensing the H_2_O_2_

**Fig. 1:**
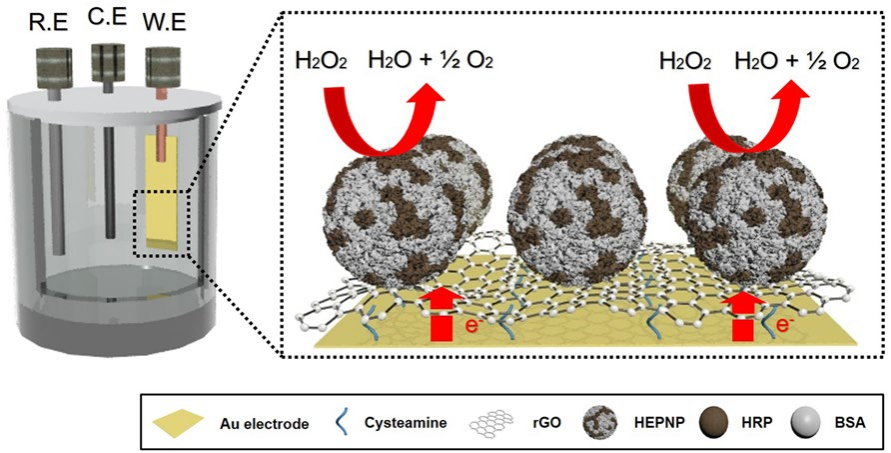
Schematic diagram of H_2_O_2_ electrochemical sensing principle of the biosensor using Horseradish peroxidase-encapsulated protein nanoparticles (HEPNP) and reduced graphene oxide (rGO) modified Au electrode. *R.E* reference electrode, *C.E* counter electrode, and *W.E* working electrode)

The fabrication method of the HEPNP/rGO/Au electrode-based sensor for the detection of H_2_O_2_ was carried out as follows: Prior to any surface modification, the Au electrode (5 × 15 mm) was cleaned using a piranha solution (H_2_SO_4_:H_2_O_2_ = 7:3) and rinsed with DW and EtOH. On the surface of the cleaned Au electrode, cysteamine (7.7 mg/mL in EtOH) acting as a chemical linker was immobilized. After cleaning the unreacted cysteamine, rGO (100 μg/mL dissolved in NMP, 50 μL) was modified on the surface of the Au electrode by an electrostatic bond between the rGO’s carboxylic functional group and the cysteamine’s amine functional group and then incubated at 75 °C. After 12 h, the rGO-immobilized Au electrode (rGO/Au) was washed with DW and dried completely. Finally, 40 μL of HEPNP was dropped on the rGO/Au electrode and incubated for 12 h at 25 °C. The final product HEPNP/rGO/Au electrode was washed using a washing solution and kept in a refrigerator at 4 °C until used. A schematic diagram of the completed HEPNP/rGO/Au working electrode’s H_2_O_2_ biosensing progress is shown in Fig. 1. H_2_O_2_ was reduced by HRP in the HEPNP, which was located on the surface of the working electrode, and the electron transfer from the Au electrode to the H_2_O_2_ through the rGO and HEPNP. This electron transfer was analyzed via the electrochemical detection technique, such as CV and amperometry i-t curve. All electrochemical tests were performed using the CHI-660e electrochemical workstation (USA) with a three-electrode system, which consisted of the working electrode, the platinum counter electrode, and a silver/silver chloride (Ag/AgCl) double-junction reference electrode.

## Results and discussion

### Characterization of the synthesized HEPNP

**Fig. 2:**
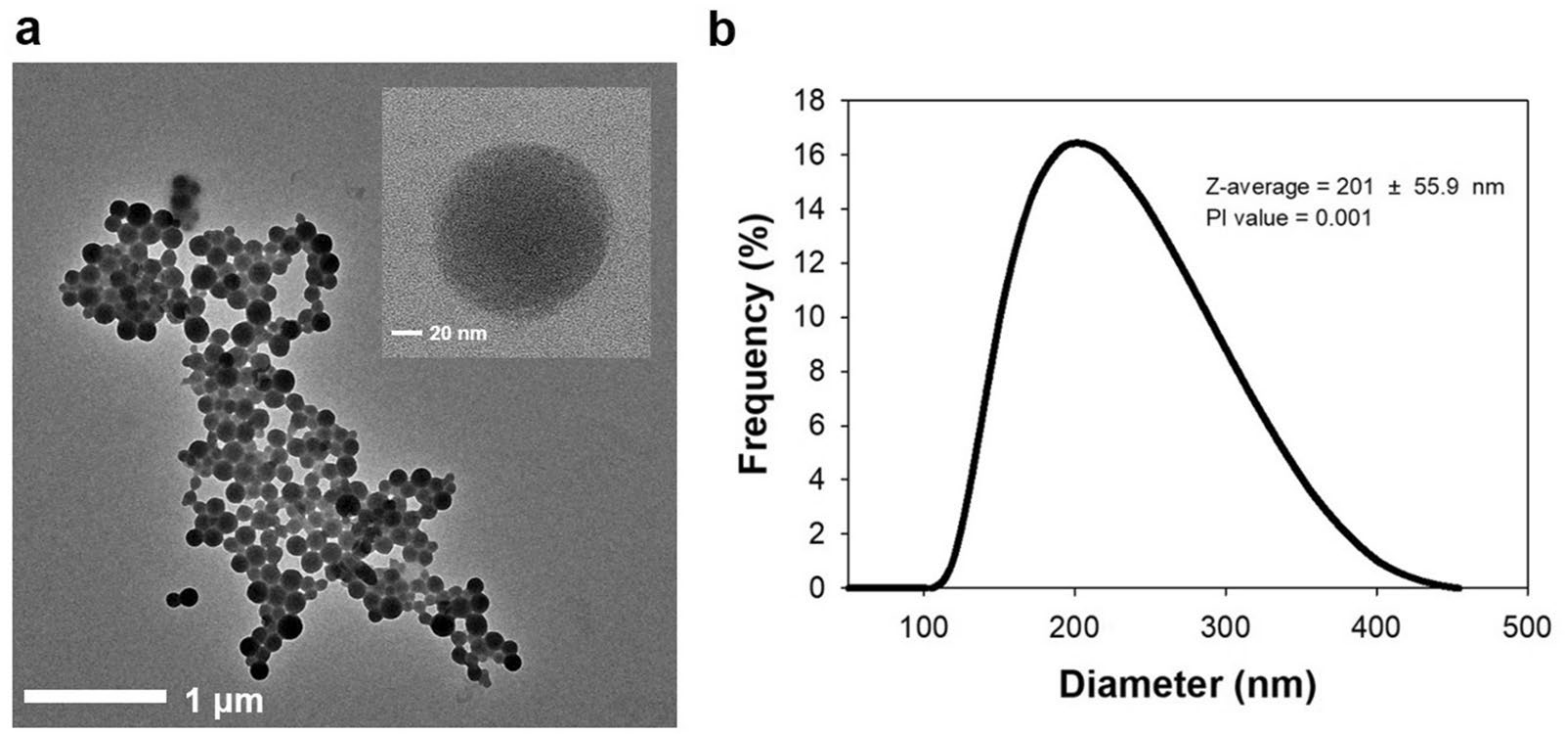
Characteristics of HEPNP. **a** Transmission electron microscopy (TEM) image of the synthesized HEPNP at pH 8, **b** Dynamic light scattering (DLS) analysis of the synthesized HEPNP at pH 8

As shown in Fig. 2a a TEM image shows the spherical morphology of an HEPNP with a diameter of about 100 nm at pH 8. The particle size of this HEPNP could be controlled by adjusting the pH value of the mixed solution, and this was due to the different molecular interactions between the proteins. This phenomenon arose from the charge of the amino acid residues, which changes depending on the pH. The spherical HEPNP indicated that HEPNP had a three-dimensional structure; therefore, the HEPNP had a larger surface area, which could hold a greater amount of HRP compared to an HRP single layer.

In our previous study, it was investigated the signal amplification of HEPNP in comparison to HRP via the enzyme–substrate reaction with a TMB substrate. The absorbance value of the same amount of HRP molecule and HEPNP, which were synthesized at pH 8, was measured. The results indicated that the HEPNP exhibited an amplified signal ca. 40 times larger than that of the HRP molecules. The significant increase in the signal of the HEPNP indicate their good applicability for the improvement of biosensor’s sensitivity [32].

To measure the particle size of HEPNP, DLS analysis was employed. As shown in Fig. 2b, the DLS results indicate that the sizes of HEPNP were distributed in the range of about 100–400 nm, the value of the Z-average was 150–250 nm, and the PI value was 0.001. The results revealed slightly different particle sizes from the results of TEM. Because DLS measures the hydrodynamic radius of the dispersed particles associated with ionic and solvent layers whereas TEM provides information about the particles’ core sizes, this cannot evaluate the particles’ hydrodynamic radius. Hence, the size obtained by DLS is usually bigger than that by TEM. In this study, to measure DLS, the HEPNP was dissolved in PBS buffer solution. Therefore, the HEPNP was swollen in the colloidal state. However, the HEPNP immobilized on the carbon-coated copper grid was in a completely dried state under vacuum conditions when using TEM.

### Surface characterization of modified electrodes

**Fig. 3:**
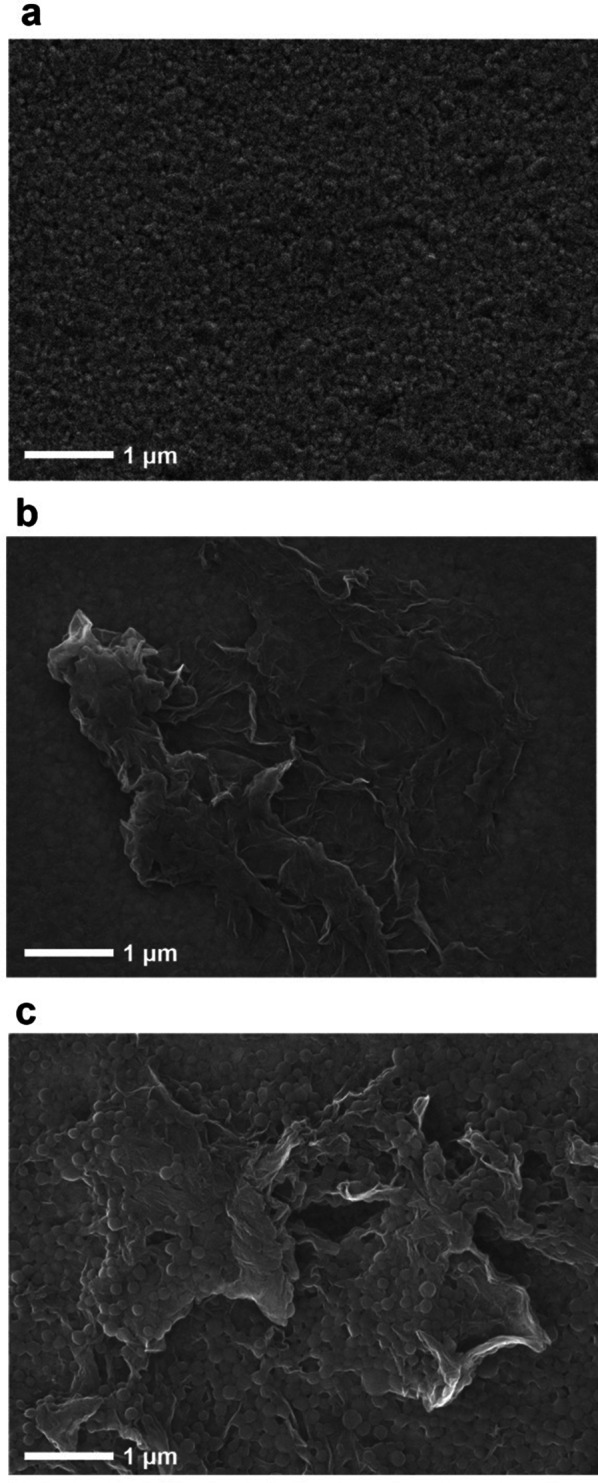
FE-SEM images of: **a** Bare Au electrode, **b** rGO/Au electrode, and **c** HEPNP/rGO/Au electrode

Figure 3a–c shows the surface morphologic properties of the working electrode, which were respectively composed of a bare Au electrode, rGO-modified Au electrode (rGO/Au), and HEPNP/rGO-modified Au electrode (HEPNP/rGO/Au), that were investigated by FE-SEM. The rGO sheets were successfully modified on the Au electrode as shown in Fig. 3b. Furthermore, it showed the morphology of the rGOs, which was formed in sheets about 3 μm in length. As shown in Fig. 3c, the HEPNP particles were tightly covered over the rGO sheets. Because the rich functional groups on the rGO surfaces enable protein molecules to efficiently adsorb onto the rGO surface, and rGO interacts with protein molecules through hydrophobic interaction, electrostatic forces, hydrogen bonding, and π-π stacking interactions occurred due to the typical sp^2^ carbon structure [33].

To compare the enzymatic activation of modified electrodes, an enzyme–substrate reaction was performed via colorimetric assay with a TMB substrate. Bare Au, HRP-immobilized Au (HRP/Au), and HEPNP-immobilized Au (HEPNP/Au) were prepared respectively, and their colorimetric signals were compared with the enzymatic reaction of the TMB substrate on each electrode surface. In the case of the HEPNP/Au electrode, it indicated a higher intensity at an absorbance 450 nm compared to the HRP/Au electrode because the HEPNP has a three-dimensional structure, which could hold a greater quantity of enzymes than am HRP single layer. Therefore, the HEPNP/Au electrode showed a powerful enzymatic reaction when using the TMB substrate (data not shown).

### Electrochemical properties of HEPNP/rGO/Au biosensor for sensitive detection of H_2_O_2_

HRP is heme-protein that can catalyze the reductive reaction of H_2_O_2_. The mechanism of electrocatalytic reduction of H_2_O_2_ is demonstrated in the following:

1 $${\text{HRP}}\left( {{\text{Fe}}^{{{\text{III}}}} } \right) \, + {\text{ H}}_{{2}} {\text{O}}_{{2}} \to {\text{ Compound I}}\left( {{\text{Fe}}^{{{\text{IV}}}} = {\text{O}},{\text{ P}}^{ \bullet + } } \right) \, + {\text{ H}}_{{2}} {\text{O}}$$

2 $${\text{Compound I}}\left( {{\text{Fe}}^{{{\text{IV}}}} = {\text{O}},{\text{ P}}^{ \bullet + } } \right) \, + {\text{ H}}_{{2}} {\text{O}}_{{2}} \to {\text{ HRP}}\left( {{\text{Fe}}^{{{\text{III}}}} } \right) \, + {\text{ O}}_{{2}}$$

3 $${\text{HRP}}\left( {{\text{Fe}}^{{{\text{III}}}} } \right) \, + {\text{ e}}^{-} \to {\text{ HRP}}\left( {{\text{Fe}}^{{{\text{II}}}} } \right){\text{ at electrode}}$$

4 $${\text{HRP}}\left( {{\text{Fe}}^{{{\text{II}}}} } \right) \, + {\text{ O}}_{{2}} \to {\text{ HRP}}\left( {{\text{Fe}}^{{{\text{II}}}} {-}{\text{ O}}_{{2}} } \right){\text{ fast}}$$

5 $${\text{HRP}}\left( {{\text{Fe}}^{{{\text{II}}}} {-}{\text{ O}}_{{2}} } \right) \, + {\text{ 2e}}^{-} + {\text{ 2H}}^{ + } \to {\text{ HRP}}\left( {{\text{Fe}}^{{{\text{II}}}} } \right) \, + {\text{ H}}_{{2}} {\text{O}}_{{2}} \;{\text{at electrode}}$$

H_2_O_2_ acts as an oxidant and a reductant in Eqs. (1) and (2), respectively, in which, HRP(Fe^III^) is oxidized by H_2_O_2_ to produce an intermediate of compound I (Fe^IV^ = O, P^•+^) first. Then, compound I is reduced by H_2_O_2_ through a two-electron transfer pathway and produces the native HRP(Fe^III^) again and O_2_. After HRP(Fe^III^) accepts one electron from the electrode to form HRP (Fe^II^) shown as Eq. (3), HRP (Fe^II^) reacts with O_2_ in a solution very rapidly to form HRP (Fe^II^–O_2_), which then undergoes electrochemical reduction at the electrode at the same potential as that of HRP (Fe^III^) reduction, producing H_2_O_2_ and HRP–Fe(^II^) again and forming a catalytic cycle with Eq. (4). H_2_O_2_ produced in Eq. (5) will participate in Eqs. (1) or (2) to produce O_2_, which will then induce or promote the catalytic cycle of Eqs. (4) and (5) [34]. Therefore, an electrochemical signal was caused by the electrocatalysis of immobilized HRP to reduce H_2_O_2_.

**Fig. 4:**
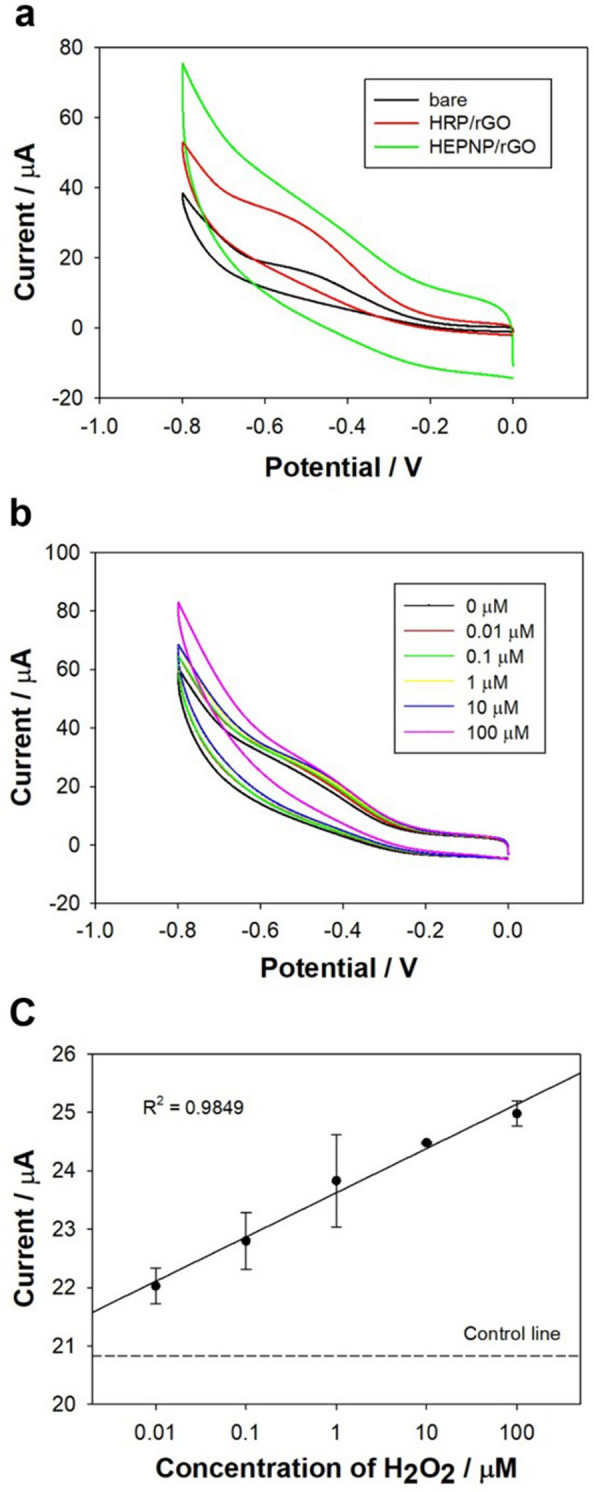
Cyclic voltammetric responses of: **a** Bare Au electrode, HRP/rGO/Au electrode, and HEPNP/rGO/Au electrode in the presence of 100 μM H_2_O_2_, **b** HEPNP/rGO/Au electrode as a function of H_2_O_2_ concentration, and **c** Linear plot of the reduction current peak at the HEPNP/rGO/Au electrode as a function of H_2_O_2_ concentration (n = 3)

The electrochemical properties of the HEPNP/rGO/Au biosensor were investigated via CV as shown in Fig. 4a–c. The parameters for CV were in the voltage range of 0 to -0.8 V, a 60 mV/s scan rate, a 1 mV/s sampling interval, 2 s of quiet time, and at a 1 × 10^−5^ (A/V) sensitivity. To confirm the electrochemical signal enhancement of the fabricated HEPNP biosensor in the detection of H_2_O_2_, the different modified electrodes were prepared respectively in the presence of 100 μM H_2_O_2_ in 0.01 M PBS solution. As shown in Fig. 4a, because of the rGO’s electrical properties, which include a good conductivity and the induction of the enhancement of electron transfer, the fabricated HRP/rGO/Au and HEPNP/rGO/Au electrode showed an increased reduction current response at − 0.45 V in comparison to the bare Au electrode. In comparison to the modified electrodes, the CV current responses of the HEPNP/rGO/Au electrode (31 μA at − 0.45 V) were higher than that of HRP/rGO/Au (24 μA at − 0.45 V), indicating that the HEPNP had enhanced the electrochemical signals in the reduction of H_2_O_2_ compared to HRP. Because the HEPNP had a greater amount of HRP, which catalyzed the reduction of H_2_O_2_, the HEPNP caused a strong enzymatic reaction with H_2_O_2_, so the electrochemical signal was enhanced. To confirm the reduction current in various scan rates, the CV of the HEPNP/rGO/Au was acquired by increasing the scan rates from 20 to 200 mV/s. Obviously, the reduction currents were enhanced with the increase of the scan rates (data not shown). To investigate the electrochemical sensitivity performance of the HEPNP biosensor in the detection of H_2_O_2_, various concentrations from 0.01–100 μM H_2_O_2_ were prepared. As shown in Fig. 4b, the reduction currents were enhanced with increases in the H_2_O_2_ concentrations. According to the CV’s responses, the detection limit of the HEPNP biosensor for H_2_O_2_ was 0.01 μM. It showed a low detection limit for H_2_O_2_ compared to other HRP-based biosensors in Table 1. The calibration curve against the various concentration of H_2_O_2_ at -0.45 V was plotted in Fig. 4c with a correlation coefficient of 0.9849. These results indicate that the fabricated HEPNP/rGO/Au biosensor had good sensitivity and could detect low concentration up to 0.01 μM of H_2_O_2_.

**Table 1:** Comparison of the electrochemical performance of HRP-based biosensors

Modified electrode	Potential (V)	Linear range (μM)	Detection limit (μM)	Reference
HRP/TB/CCB	− 0.25	0.429–455	0.17	[35]
HRP-MIL100(Cr)-B	− 0.3	0.5–3000	0.1	[36]
HRP-BMIM-BF_4_/SWCNTs/CFUME	− 0.35	0.49–10.2	0.13	[37]
Flower-like Bi_2_WO_6_	− 0.35	0.5–100	0.18	[38]
HRP-MoS_2_-Gr	− 0.08	0.2–1.103	0.049	[39]
Co_3_O_4_/MWCNTs/gelatin/HRP	− 0.3	0.74–19	0.74	[40]
HEPNP/rGO/Au electrode	− 0.45	0.01–100	0.01	Present work

*TB* toluidine blue, *CCB* ceramic composite biosensor, *MIL100(Cr)-B* mil100(cr)-b boronic acid functionalized metal–organic frameworks, *BMIM* 1-butyl-3-methylimidazolium tetrafluoroborate, *SWCNTs* Single-walled carbon nanotubes, *CFUME* Carbon fiber ultramicroelectrodes, *MoS*_*2*_ molybdenum disulfide gr graphene, *Co*_*3*_*O*_*4*_ cobalt oxide, *MWCNTs* multiwall carbon nanotubes, *HEPNP* horseradish peroxidase encapsulated protein nanoparticles, *rGO* reduced graphene oxide Au: gold electrode

### Selective amperometry response of the HEPNP/rGO/Au electrode in the detection of H_2_O_2_

**Fig. 5:**
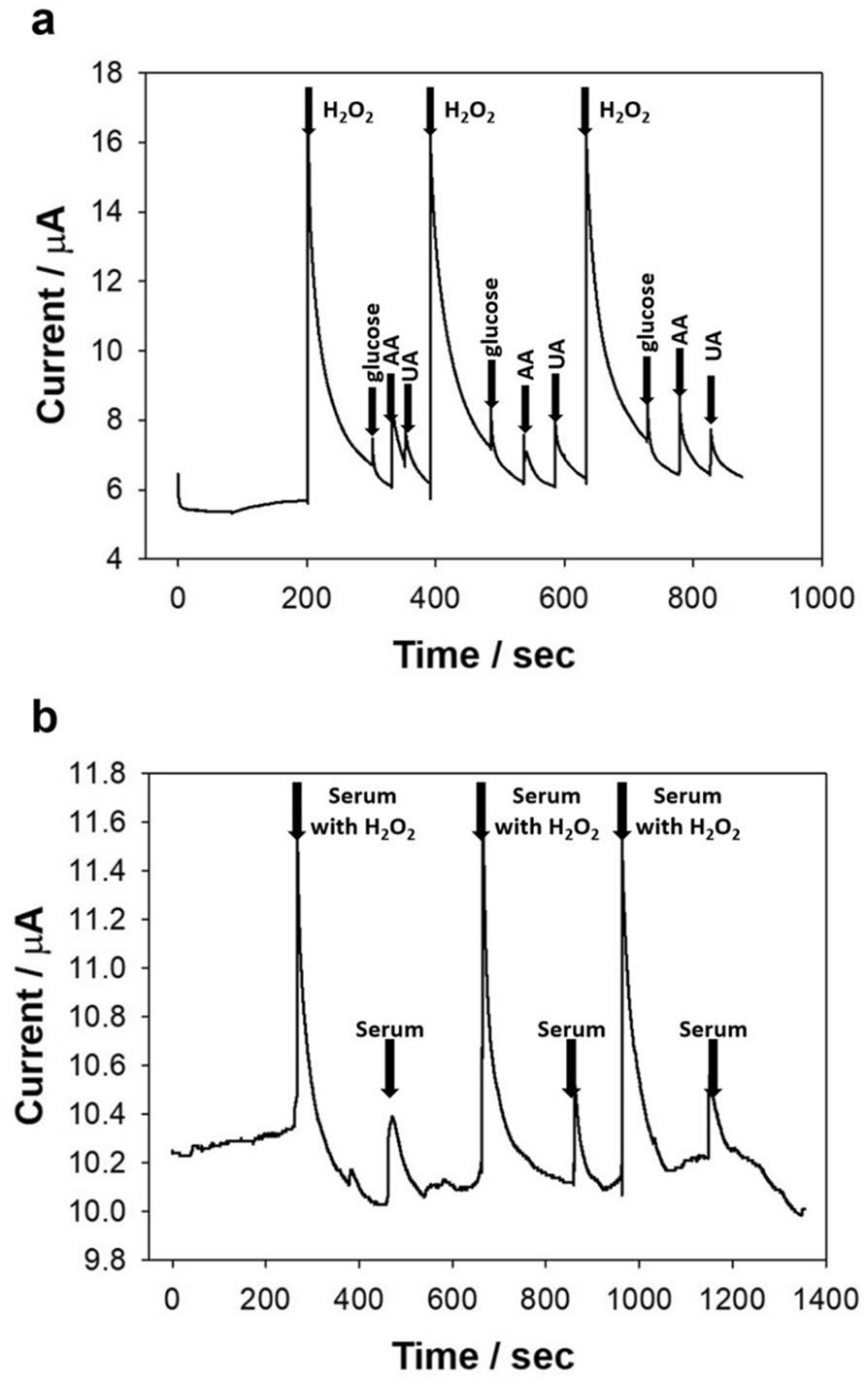
Amperometric responses of the HEPNP/rGO/Au electrode for: **a** H_2_O_2_, glucose, ascorbic acid, and uric acid at the same concentration (100 μM) in PBS buffer and **b** H_2_O_2_ (10 μM) in human serum

To test the selectivity performance of the HEPNP/rGO/Au electrode to H_2_O_2_, some co-existing species in bio-fluids, such as glucose, UA, and AA, were analyzed via the amperometry response with H_2_O_2_. Parameters for i-t curve amperometry analysis were a 0.1 s sampling interval, 0 s quiet time, 1 × 10^−5^ (A/V) sensitivity, and -0.45 V as the initial voltage. All samples were prepared at a final concentration of 100 μM on PBS. Figure 5a shows that the response current was increased to be much higher in the case of H_2_O_2_ addition than the results of glucose, AA, and UA addition. It can be seen that this fabricated biosensor showed highly selective performance in the amperometric response of H_2_O_2_ towards glucose, UA, and AA.

To verify the sensing performance of H_2_O_2_ by the HEPNP/rGO/Au electrode sensor in the actual serum sample, an experiment was conducted with a human blood serum sample. After 200 s of signal stabilization, 20 μL of serum with 10 μM H_2_O_2_ was added to 5 mL of 0.01 M PBS solution, and 20 μL of serum without H_2_O_2_ was added to 5 mL of 0.01 M PBS solution three times. Figure 5b shows the high response current when using serum with H_2_O but a weak response current when using serum without H_2_O_2_. In addition, it can also be confirmed that H_2_O_2_ in the serum can be measured using a constant signal. These results demonstrate that the HEPNP/rGO/Au**-**based electrochemical biosensor can selectively detect H_2_O_2_ in human serum with stable response and can be applied to clinical sample analysis.

## Conclusion

In this study, an electrochemical biosensor composed of HEPNP was fabricated for highly sensitive and selective H_2_O_2_ detection. In this biosensor, rGO was used to increase efficiency of electron transfer from Au electrode to HEPNP, and HEPNP was used for increasing sensitivity by fixing a great amount of HRP with a three-dimensional structure on the surface of electrode. The results of the electrochemical investigation suggest that the HEPNP/rGO/Au biosensor results in a highly enhanced electrochemical signal compared to using an HRP/rGO/Au. This biosensor could operate linearly with different concentrations of H_2_O_2_ and could detect low concentrations up to 0.01 μM. Furthermore, this biosensor had a good selectivity for the detection of H_2_O_2_ and could detect H_2_O_2_ dissolved in human serum. Consequently, this proposed HEPNP/rGO/Au biosensor has a wide range of possible applications in the measurement of very small quantities of H_2_O_2_ in the chemical, biological, and clinical fields.

## Data Availability

The datasets used and/or analyzed during the current study are available from the corresponding author on reasonable request.

## Abbreviations

H_2_O_2_: Hydrogen peroxide
HRP: Horseradish peroxidase
BSA: Bovine serum albumin
HEPNP: HRP encapsulated protein nanoparticles
Au: Gold electrode
rGO: Reduced graphene oxide
CV: Cyclic voltammetry
ROS: Reactive oxygen species
TEM: Transmission electron microscopy
DLS: Dynamic light scattering
FE-SEM: Field emission scanning electron microscope
PBS: Phosphate bufferd saline
TMB: 3,3′,5,5′-Tetramethylbenzidine
AA: L-ascorbic acid
UA: Uric acid
NMP: N-methyl-2-pyrrolidone
DW: Distilled water

## References

[1] Chang SC (2017). J. Food Hyg. Saf..

[2] Halliwell B, Clement MV, Long LH (2000). FEBS Lett..

[3] Yagati AK, Choi JW (2014). Electroanalysis.

[4] Chen W, Cai S, Ren QQ, Wen W, Zhao YD (2012). Analyst.

[5] Eich RF, Li TS, Lemon DD, Doherty DH, Curry SR, Aitken JF, Mathews AJ, Johnson KA, Smith RD, Phillips GN, Olson JS (1996). Biochemistry.

[6] Marzo ND, Chisci E, Giovannoni R (2018). Cells.

[7] Ishikawa K, Takenaga K, Akimoto M, Koshikawa N, Yamaguchi A, Imanishi H, Nakada K, Honma Y, Hayashi J (2008). Science.

[8] Gaschler MM, Stockwell BR (2017). Biochem. Biophys Res. Commun..

[9] Dalle-Donne I, Giustarini D, Colombo R, Rossi R, Milzani A (2003). Trends Mol Med..

[10] Cadenas E, Davies KJA (2000). Free Radic Biol Med..

[11] Molaabasi F, Hosseinkhani S, Moosavi-Movahedi AA, Shamsipur M (2015). Rsc Adv..

[12] Cocheme HM, Quin C, McQuaker SJ, Cabreiro F, Logan A, Prime TA, Abakumova I, Patel JV, Fearnley IM, James AM, Porteous CM, Smith RAJ, Saeed S, Carre JE, Singer M, Gems D, Hartley RC, Partridge L, Murphy MP (2011). Cell Metab..

[13] Jia Y, Sun S, Cui X, Wang X, Yang L (2019). Talanta.

[14] Shamsipur M, Pashabadi A, Molaabasi F (2015). RSC Adv..

[15] Kurowska E, Brzozka A, Jarosz M, Sulka GD, Jaskula M (2013). Electrochim. Acta..

[16] Yoon J, Lee T, Bapurao GB, Jo J, Oh BK, Choi JW (2017). Biosens. Bioelectron..

[17] Yang L, Xu C, Ye W, Liu W (2015). Sens. Actuators B Chem..

[18] Kong YT, Boopathi M, Shim YB (2003). Biosens Bioelectron..

[19] Geim AK, Novoselov KS (2007). Nat Mater..

[20] Novoselov KS, Jiang Z, Zhang Y, Morozov SV, Stormer HL, Zeitler U, Maan JC, Boebinger GS, Kim P, Geim AK (2007). Science.

[21] Lee C, Wei X, Kysar JW, Hone J (2008). Science.

[22] Nandini S, Nalini S, Manjunatha R, Shanmugam S, Melo JS, Suresh GS (2013). J Electroanal Chem.

[23] Nalini S, Nandini S, Shanmugam S, Neelagund SE, Melo JS, Suresh GS (2014). J Solid State Electrochem..

[24] Zhou K, Zhu Y, Yang X, Luo J, Li C, Luan S (2010). Electrochim Acta..

[25] Zhou Z, Hartmann M (2013). Chem Soc Rev..

[26] Cao X, Li Y, Zhang Z, Yu J, Qian J, Liu S (2012). Analyst.

[27] Yu J, Zhang Y, Liu S (2014). Biosens Bioelectron..

[28] Wang Y, Chen X, Zhu J (2009). Electrochem Commun..

[29] Dai M, Huang T, Chao L, Xie Q, Tan Y, Chen C, Meng W (2016). Talanta.

[30] Chen W, Yang W, Lu Y, Zhu W, Chen X (2017). Anal Methods..

[31] Bai G, Xu X, Dai Q, Zheng Q, Yao Y, Liu S, Yao C (2019). Analyst.

[32] Choi JH, Lim YT, Oh BK (2014). Sci Adv Mater..

[33] Huang Y, Hara A, Terashima C, Fujishima A, Takai M (2019). Carbon.

[34] Zhao H, Sheng Q, Zheng J (2012). Microchim Acta..

[35] Thenmozhi K, Narayanan SS (2017). Mater Sci Eng C..

[36] Dai H, Lu W, Zuo X, Zhu Q, Pan C, Niu X, Liu J, Chen H, Chen X (2017). Biosens Bioelectron..

[37] Ren QQ, Wu J, Zhang WC, Wang C, Qin X, Liu GC, Li ZX, Yu Y (2017). Sens Actuators B Chem..

[38] Liu H, Guo K, Duan C, Chen X, Zhu Z (2016). Mater Sci Eng C..

[39] Song H, Ni Y, Kokot S (2014). Biosens Bioelectron..

[40] Kacar C, Dalkiran B, Erden PE, Kilic E (2014). Appl Surf Sci..

